# Clinical experience with emapalumab treatment in a group of children with primary hemophagocytic lymphohistiocytosis

**DOI:** 10.70962/jhi.20250122

**Published:** 2025-12-11

**Authors:** Yulia Rodina, Vasiliy Burlakov, Varvara Brilliantova, Anna Roppelt, Nelli Kan, Ulijna Petrova, Elena Raykina, Dmitry Pershin, Dmitry Balashov, Alexey Maschan, Galina Novichkova, Anna Shcherbina

**Affiliations:** 1 Dmitry Rogachev National Medical Research Center of Pediatric Hematology, Oncology and Immunology, Department of Immunology, Moscow, Russia; 2 Moscow City Hospital 52, Moscow, Russia

## Abstract

Primary hemophagocytic lymphohistiocytosis (pHLH) is a group of genetically determined disorders characterized by severe and fulminant systemic inflammation, cytopenia, and multiple organ involvement. A dexamethasone-and-etoposide–based HLH-2004 protocol is widely used to treat pHLH, yet often fails to produce the disease control required for the next treatment step: hematopoietic stem cell transplantation (HSCT). We report on the use of the interferon gamma inhibitor emapalumab in seven children aged 6–39 months with pHLH. Three patients received emapalumab at an average starting dose of 1.7 mg/kg and had no active HLH by median day 28. Four patients received emapalumab at an average starting dose of 7.2 mg/kg and had no active HLH by median day 14 (P = 0,0015). We suggest that the higher starting dose of emapalumab, as well as its combination with Janus kinase inhibitors, might increase the remission rate in pHLH and the success of subsequent HSCT.

## Teaser

This study explores the use of emapalumab, an interferon gamma inhibitor, in seven children with primary hemophagocytic lymphohistiocytosis. Higher initial doses of emapalumab and combination therapy with JAK inhibitors significantly accelerated disease remission, potentially improving outcomes and the success of subsequent hematopoietic stem cell transplantation.

## Introduction

Primary hemophagocytic lymphohistiocytosis (pHLH) or familial hemophagocytic lymphohistiocytosis (FHLH) (1) encompasses a group of genetically determined disorders characterized by severe, often fulminant systemic inflammation, cytopenia, and multiple organ involvement. The disease manifests predominantly in early childhood, has a high mortality rate, and requires hematopoietic stem cell transplantation (HSCT) as the only currently available curative option (2). However, HSCT outcomes are dependent on the state of remission of the underlying systemic inflammation and the infection complications, as well as organ damage due to the side effects of any chemotherapeutic drugs received prior to HSCT (3). For years, the gold standard for HLH treatment has been the dexamethasone-and-etoposide–based HLH-2004 protocol (4), yet it has failed to uniformly control the disease (5). An alternative regimen that combines antithymocyte globulin and corticosteroids has demonstrated good results but has barely improved survival (6). In addition, no universally accepted treatment exists for relapsed or refractory HLH. Advances in the availability of biological treatments have opened new possibilities for HLH therapy, with several targets proposed in recent years. The interleukin-receptor-6 antagonist tocilizumab, the interleukin-1 inhibitor anakinra, and the Janus kinase inhibitor (JAKinib) ruxolitinib have been used for HLH treatment, with variable results (7, 8, 9).

Mounting evidence has provided support for the pivotal pathogenic role of interferon-γ (IFNγ) in HLH (10). Emapalumab is a fully human IgG1 anti-IFNγ monoclonal antibody that binds free and receptor-bound IFNγ and inhibits its biologic activity. The data accumulated from phase II and III clinical trials and reports regarding small groups of patients demonstrate emapalumab efficacy in pHLH (11, 12, 13, 14, 15). Herein, we describe our experience with emapalumab treatment in a cohort of 7 pediatric patients with pHLH.

## Results

The study included seven patients (four male, three female) with various forms of pHLH (three with X-linked lymphoproliferative syndrome type 2 [XLP2], two with FHLH3, and two with FHLH1) (Table 1). The age of diagnosis was 7 mo (Me, 1;37), and the age at the start of emapalumab treatment was 8 mo (Мe, 6;39). All patients had active HLH at the start of emapalumab treatment, including two with central nervous system (CNS) involvement, which was diagnosed based on magnetic resonance imaging results and cerebrospinal fluid examination. Two had first ongoing first episodes of HLH refractory to treatment with dexamethasone and VP-16, and five had HLH relapses.

**Table 1.** Patients’ characteristics

Patients/gender	Gene/variants	Age of HLH onset/emapalumab start (mo)	Infections prior to emapalumab	HLH treatment at the start of emapalumab	Reason for emapalumab/dose (mg/kg/twice a week)	HLH treatment at the end of emapalumab	Type of donor/transplant processing	Conditioning regimen/GVHD prophylaxis	Outcome/HLH status
P1/M	*XIAP* (NM_001167.4): c.612_614del, p.Gly205del, hemi	38/39	Pneumonia, CMV infection	Dexa 10 mg/m^2^ VP 150 mg/m^2^/weekly Ruxo 30 mg/m^2^	Relapse/10	Dexa 3 mg/m^2^ VP 150 mg/m^2^/weekly Ruxo 30 mg/m^2^	Haplo, TCRαβ+/CD19+ depletion	Аtgam 5 mg/kg Mel 180 mg Rit 200 mg TLI 4 Gy Flu 150 mg/m^2^ Cyc 120 mg/m^2^/*Ruxo*	Died (post-HSCT complications)
P2/M	*XIAP* (NM_001167.4): c.1027_1030del, p.(His343_Leu344delinsTer), hemi	6/7	Pneumonia, CMV infection	Dexa 10 mg/m^2^ Ruxo 25 mg/m^2^ Toci 10 mg/2 wk	No remission/1.3–3.3	Dexa 0 mg/m^2^ Ruxo 25 mg/m^2^	MUD (10/10), TCRαβ+/CD19+ depletion	TT 10 mg/kg Treo 36 mg/m^2^ Flu 150 mg/m^2^ ATG 5 mg/kg Rit 100 mg/*Ruxo*	36 mo after HSCT/HLH remission
P3/F	*UNC13D* (NM_199242.3): c.2258_2276delinsTACCTTGTCCGA, p.(Gly753ValfsTer40), homo	1/6	Enterocolitis (*Klebsiella pneumoniae* ESBL, norovirus)	HLH-2004 Toci 10 mg/2 wk	Relapse/1.5	Dexa 0 mg/m^2^ VP 150 mg/m^2^/2 wk Toci 10 mg/2 wk CsA	Haplo, TCRαβ+/CD19+ depletion	TT 10 mg/kg Treo 36 mg/m^2^ Flu 150 mg/m^2^ ATG 5 mg/kg Rit 50 mg/*CsA, abatacept*	28 mo after HSCT/HLH remission
P4/F	u/n	9/18	Pneumonia, Enterocolitis (norovirus)	Dexa 10 mg/m^2^ Toci 10 mg/2 wk Bari 4 mg/daily Anakinra 10 mg/kg/daily SQ	Relapse/10-6-1	Dexa 0.5 mg/m^2^ Bari 4 mg/daily Anakinra 10 mg/kg/daily SQ	No HSCT	-	Lost to follow-up
P5/M	*XIAР* (NM_001167.4): del 2–5 ex, hemi	7/8	Pneumonia	Dexa 10 mg/m^2^ VP 150 mg/m^2^/weekly Ruxo 30 mg/m^2^ Toci 10 mg/2 wk	Relapse/6–1.2	Dexa 0 mg/m^2^ Ruxo 30 mg/m^2^	Haplo, TCRαβ+/CD19+ depletion	TT 10 mg/kg Treo 36 mg/m^2^ Flu 150 mg/m^2^ ATG 5.5 mg/kg Rit 100 mg/*Ruxo*	34 mo after HSCT/HLH remission
P6/F	u/n	11/12	No	Dexa 10 mg/m^2^ VP 150 mg/m^2^/weekly MTX + dexa IT	No remission/10–1.5	Dexa 0.35 mg/m^2^ VP 150 mg/m^2^/2 wk MTX + dexa IT	Haplo, TCRαβ depletion	TT 10 mg/kg Treo 42 mg/m^2^ Flu 150 mg/m^2^ ATG 5 mg/kg Rit 100 mg/*abatacept*	Died (post-HSCT complications)
P7M	*UNC13D* (NM_199242.3): c.2346_2349del, p.(Arg782SerfsTer12); c.3011T>C, p.(Leu1004Pro), compound het	5/6	Perianal necrotizing dermatitis (*Klebsiella pneumoniae* ESBL, *Enterobacter asburiae* ESBL)	HLH-2004 MTX + dexa IT	Relapse/1.5	Dexa 1.25 mg/m^2^ VP 150 mg/m^2^/2 wk CsA MTX + dexa IT	Haplo, TCRαβ+/CD19+ depletion	Аtgam 5 mg/kg Treo 36 mg/m^2^ Flu 150 mg/m^2^ Rit 100 mg/*CsA, abatacept*	20 mo after HSCT/HLH remission

Atgam, anti-T lymphocyte immunoglobulin for human use, animal (equine); Mel, melphalan; Rit, rituximab; TLI, total lymphoid irradiation; Flu, fludarabine; Cyc, cyclophosphamide; CsA, cyclosporine; TT, thioTEPA; Treo, treosulfan; ATG, anti-T lymphocyte immunoglobulin for human use, animal (rabbit); GVHD, graft-versus-host disease; VP, etoposide; MTX, methotrexate; Dexa, dexamethasone; Toci, tocilizumab; Ruxo, ruxolitinib; Bari, baricitinib; IT, intrathecally; CMV, cytomegalovirus; ESBL, extended spectrum β-lactamase. The text in italics indicates the medications used for the prophylaxis of acute graft-versus-host disease (GVHD).

Preceding/concurrent immunosuppressive therapy included variations of the HLH-2004 protocol, with the addition of tocilizumab in three patients, JAKinibs in four, and anakinra in one. Two patients with CNS involvement received methotrexate and dexamethasone intrathecally (Table 1).

Six patients had prior or ongoing infectious episodes while on immunosuppressive treatment (Table 1).

Infectious complications that had developed since the onset of HLH and before emapalumab initiation were caused predominantly by fungal and bacterial pathogens and included pneumonia in four patients, enterocolitis in four, and perianal necrotizing dermatitis in one (Table 1). In three of the seven patients, severe viral infections occurred, including cytomegalovirus disease in two and enterocolitis caused by norovirus in two.

Other complications of HLH treatment included secondary Cushing syndrome in all seven patients, osteoporosis in one, hypertension in three, seizures due to cyclosporine toxicity in one, and kidney damage in one.

Emapalumab was administered twice weekly for a median of 6.3 wk (2–13 wk). In the first three patients, emapalumab was administered in a rump-up fashion at an average starting dose of 1.7 mg/kg (1.2–3.3 mg/kg), with the average highest dose being 3.3 mg/kg (low-dose group). The next four patients received emapalumab in a step-down fashion at an average starting dose of 7.2 mg/kg (6–10 mg/kg), with the average lowest dose being 1.2 mg/kg (high-dose group) (Table 1). P6 received two courses of emapalumab due to a relapse of HLH prior to HSCT. The analysis of efficacy included only the first course of treatment, while the analysis of safety included all emapalumab infusions.

After 2 wk of emapalumab therapy in both groups, significant clinical and laboratory improvement was documented (P < 0.05), as follows: ferritin and triglyceride levels decreased from 4,480 ± 3,100 to 825 ± 282 ug/L and from 3.58 ± 1 to 1.7 ± 0.2 mM/L, respectively; fibrinogen increased from 1.4 ± 0.2 to 2.3 ± 0.2 g/L. Platelet and neutrophil counts increased from 180 ± 70 × 10^9^/L to 237 ± 40 × 10^9^/L and from 1.0 ± 0.6 × 10^9^/L to 2.3 ± 1.0 × 10^9^/L, respectively; and hemoglobin increased from 106.5 ± 12 to 111 ± 13 g/L (Table 2). Hepatosplenomegaly was mostly resolved by week 4 in five of the six patients with this symptom.

**Table 2.** Dynamics of laboratory parameters during emapalumab therapy

Parameters, mean ± SEM	Before emapalumab therapy	After emapalumab therapy	Normal range
Ferritin, µg/L	4,480 ± 3,100	825 ± 282	20–140
Triglyceride, mM/L	3.58 ± 1	1.7 ± 0.2	0–2.3
Fibrinogen, g/L	1.4 ± 0.2	2.3 ± 0.2	2–3.93
Platelet, ×10^9^/L	180 ± 70	237 ± 40	204–356
Neutrophil counts, ×10^9^/L	1.0 ± 0.6	2.3 ± 1.0	2.27–5.56
Hemoglobin, g/L	106.5 ± 12	111 ± 13	115–138

**Figure 1.**
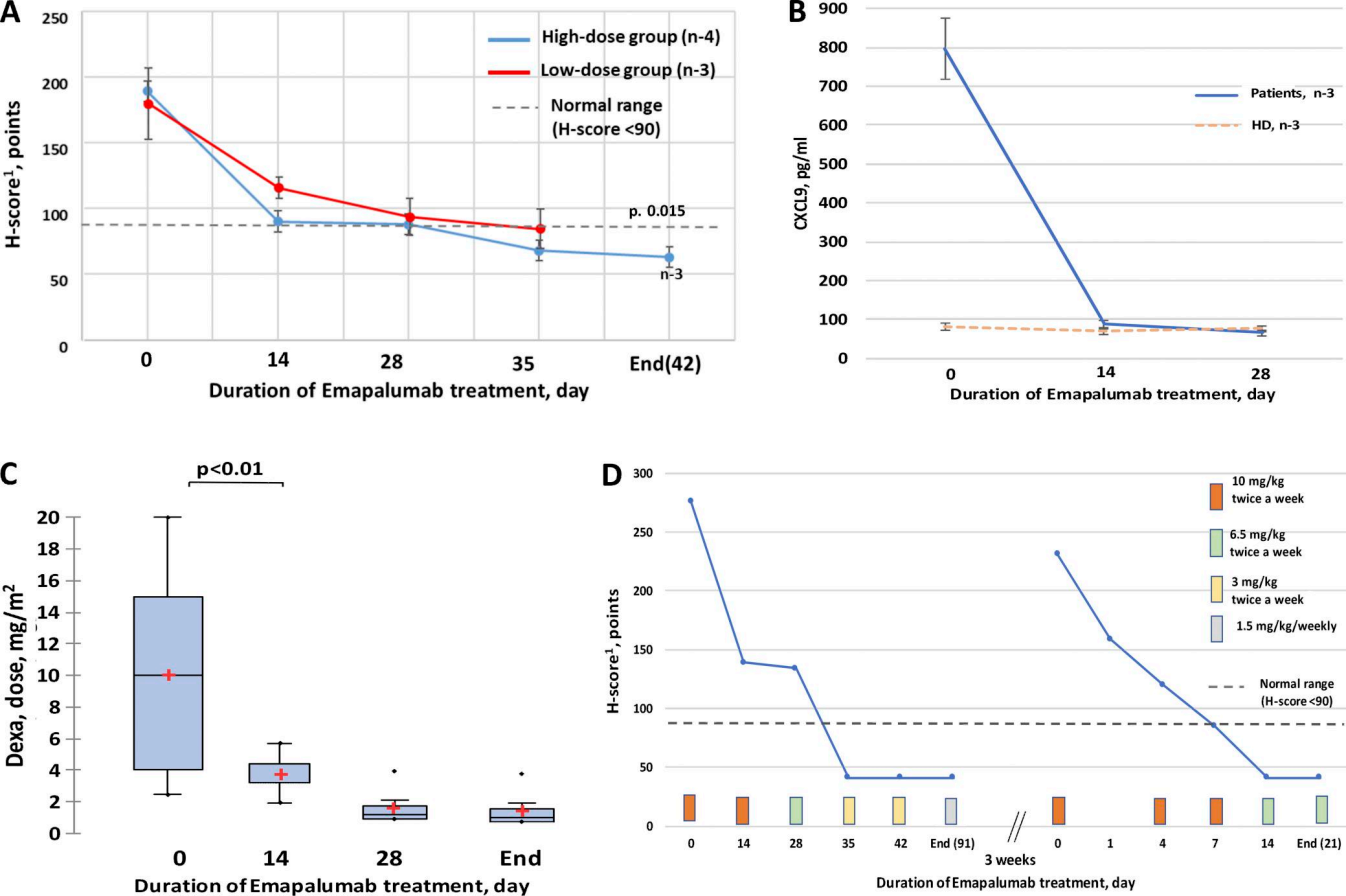
**Dynamics of clinical and laboratory parameters in pHLH patients during emapalumab treatment. (A)** H-score decrease in low- and high-dose emapalumab groups. H-score of 90 was used as a cutoff for active disease. **(B)** Dynamics of CXCL9 level on emapalumab therapy. **(C)** Dynamics of dexamethasone dose reduction on emapalumab therapy. **(D)** Dynamics of HLH activity in P6, who had a relapse of the disease after emapalumab discontinuation and quickly responded to emapalumab treatment reinstitution.

All patients had no active disease by the end of their treatment periods. Yet, in the high-dose group, two of the four patients reached no active disease status by day 14, and three of the four by day 28. In the low-dose group, disease was still active in all on day 14; one of the three patients achieved no active disease status by day 28 (Fig. 1 A). Time to no active disease status was significantly shorter in the high-dose group—Ме 14 days (14;28), than in the low-dose group—Ме 28 days (28–35) (P = 0.0015).

IFNγ-induced chemokine CXCL9 level has been shown to correlate with HLH activity (16). In the entire cohort, there was an eightfold decrease in CXCL9 levels by day 28 of treatment (Fig. 1 B). Treatment with emapalumab enabled a reduction of the dexamethasone dose from 10 ± 2 (mg/m^2^) at baseline to 0.7 ± 0.4 mg/kg/day by week 4 (Fig. 1 C), as well as discontinuation of some of the drugs used for the HLH treatment (Table 1).

P6 started at 10 mg/kg/day of emapalumab and had no active HLH by week 4 (H-score 88). Her dose was gradually decreased to 1.5 mg/kg/twice weekly, and therapy was discontinued at week 13, although the patient continued to receive maintenance therapy with dexamethasone and etoposide according to the HLH-2004 protocol (H-score 47). By week 16, 3 wk after discontinuing emapalumab, an HLH relapse was documented, defined as having an H-score of 231. The patient was restarted on 10 mg/kg/twice weekly of emapalumab and achieved no active disease status (H-score 85) after only three doses. She then continued on 6 mg/kg/twice weekly of emapalumab until day +7 after HSCT (see below, Fig. 1 D).

Eventually, six of the seven patients underwent HSCT without relapses of HLH in the posttransplant period (Table 1). One patient’s family refused HSCT, and she was lost to the study’s follow-up. In addition to P6, two more transplanted patients continued emapalumab until day −1 before HSCT, when it was discontinued.

Two patients died after HSCT. P1 developed grade 4 graft-versus-host-disease, involving the skin, liver, and intestines and including progressive transplant hypofunction and multiorgan failure, which resulted in death at day 74 after HSCT. This patient, who belonged to the high-dose group, had stopped emapalumab infusions 30 days prior to HSCT.

P6 failed to achieve engraftment after the first HSCT. Three months later, she received a second HSCT, which was complicated by liver veno-occlusive disease, severe infectious complications, and progressive organ failure, which resulted in death at day +99 after second HSCT.

The patients received a total of 72 infusions of emapalumab. No side effects or toxicity were noted during the treatment, nor were any new infectious episodes or worsening of preexisting ones documented during the treatment.

## Discussion

Emapalumab is the first cytokine-targeting preparation to be approved by the U.S. Food and Drug Administration specifically for the treatment of HLH (17).

Based on our experience with emapalumab in a previously treated cohort of pHLH children, we conclude that it is effective in controlling HLH manifestations and safe for pediatric patients. In our group, all patients achieved control of HLH, and the overall survival rate, accounting for HSCT outcomes, was 71%. This is comparable to the results of the clinical trial described by Locatelli et al. (11). However, in that trial, 37% of previously treated patients had no response to emapalumab, whereas in our small group, all patients achieved no active disease status.

It is worth noting that four patients received emapalumab together with JAKinibs, a combination previously reported to be safe and effective in a patient with Epstein-Barr virus–associated HLH (18). The use of JAKinibs and other anticytokine monoclonal antibodies in combination with emapalumab might explain the superior results in our group of patients. Based on these experiences, we feel that this drug combination needs to be studied in larger groups of primary and secondary HLH patients and might become the cornerstone of a new protocol for treating resistant disease. We also demonstrated that patients who received a higher starting dose of emapalumab achieved no active disease status faster, and this fact might have contributed to the overall success of the treatment prior to HSCT in our cohort.

Another issue that needs to be explored further is the duration of emapalumab treatment in the HSCT setting. One patient in the above-mentioned study experienced a relapse of HLH before HSCT after emapalumab treatment was stopped (18). In our cohort, one patient experienced a relapse of HLH after elective emapalumab discontinuation prior to HSCT, but her symptoms were quickly ameliorated after emapalumab was reintroduced. Prolonged IFNγ inhibition might not only have prevented HLH flares before or shortly after HSCT but also have reduced rejection rates in this difficult cohort of patients, considering that IFNγ has been implicated in graft rejection in various clinical models (19). A recent study by Verkamp et al. compared a group of 22 patients with pHLH who received emapalumab for up to 21 days prior to conditioning with a group of 28 patients who did not receive the drug. The emapalumab group demonstrated a markedly lower incidence of mixed chimerism, higher intervention-free survival, and a tendency toward improved overall survival (20). Most of our patients stopped emapalumab shortly before HSCT. P6, who had the highest score of all at the beginning of emapalumab treatment and relapsed after its discontinuation, had the most severe disease in our group. She continued the treatment until day +7 after HSCT, yet failed to engraft. Since cytokine surge can last until 30 days after HSCT (21), we hypothesized that longer IFNγ might have been beneficial for her and other pHLH patients. Retrospective nature, small group size, and variable treatment protocols constitute limitations of this study, yet we feel that several questions raised by us require further investigation in larger prospective studies.

Recently, the use of emapalumab has been extended to patients with secondary HLH or macrophage activation syndrome (22, 23). Therefore, use of emapalumab in primary and secondary HLH represents a shift away from prolonged nonspecific cytotoxic chemotherapy and toward more targeted immune modulation. The issue of treatment duration and the algorithms of dose tapering remain to be studied in larger cohorts of patients, especially with secondary HLH patients who are not considered as candidates for HSCT. More advanced biological markers of HLH remission or relapse, such as CXCL9, are crucial to establishing these protocols.

In conclusion, we demonstrate the safety and efficacy of emapalumab in a cohort of children with pHLH and propose further studies of dosing regimens and treatment duration.

## Materials and methods

We conducted a retrospective study assessing the efficacy and safety of emapalumab in children with pHLH who received treatment at our center between November 2019 and September 2024. The study was conducted in accordance with the Helsinki Declaration and approved by the local ethics committee, and the patients’ parents gave written consent for treatment and participation in the study.

The study was registered at ClinicalTrials.gov (ID: NCT06587191).

The inclusion criterion was a diagnosis of pHLH according to the criteria of the Histiocytic Society (4). Genetic variant identification utilized high-throughput next-generation sequencing using custom gene panels designed specifically for IEI diagnostics. The gene composition is available upon request. If two patient-relevant genetic variants were not found, their material has been sent for whole-genome sequencing (pending). In them, the diagnosis of pHLH was made based on the early age of onset, no obvious causes of secondary HLH, and laboratory test, including low natural killer cytotoxicity. The severity of the disease was assessed using an adapted H-score (24) with the exclusion of bone marrow hemophagocytosis, and for the purpose of current study, “no active disease” status was defined as an H-score < 90.

The concentration of CXCL9, which is the marker of HLH activity, in the patients’ previously frozen and stored serum was assessed using the Luminex Magpix Multiplex Assay system (Bio-Rad) and a Luminex Human Magnetic Assay kit (R&D Systems) according to the manufacturers’ protocols.

Statistical analysis was conducted using Addinsoft 2020 (XLSTAT). Fisher’s exact test was used to compare categorical variables. Statistical significance was set at P ≤ 0.05.

## Data Availability

Data are available in the article itself and its supplementary materials. The data underlying Fig. 1 and Tables 1 and 2 are available in the published article.

## References

[1] Canna, S.W., and R.A. Marsh. 2020. Pediatric hemophagocytic lymphohistiocytosis. Blood. 135:1332–1343. 10.1182/blood.2019000936 32107531 PMC8212354

[2] Ehl, S., I. Astigarraga, T. von Bahr Greenwood, M. Hines, A. Horne, E. Ishii, G. Janka, M.B. Jordan, P. La Rosée, K. Lehmberg, . 2018. Recommendations for the use of etoposide-based therapy and bone marrow transplantation for the treatment of HLH: Consensus statements by the HLH steering committee of the histiocyte society. J. Allergy Clin. Immunol. Pract. 6:1508–1517. 10.1016/j.jaip.2018.05.031 30201097

[3] Allen, C.E., R. Marsh, P. Dawson, C.M. Bollard, S. Shenoy, P. Roehrs, R. Hanna, L. Burroughs, L. Kean, J.A. Talano, . 2018. Reduced-intensity conditioning for hematopoietic cell transplant for HLH and primary immune deficiencies. Blood. 132:1438–1451. 10.1182/blood-2018-01-828277 29997222 PMC6161764

[4] Henter, J.I., A. Horne, M. Aricó, R.M. Egeler, A.H. Filipovich, S. Imashuku, S. Ladisch, K. McClain, D. Webb, J. Winiarski, and G. Janka. 2007. HLH-2004: Diagnostic and therapeutic guidelines for hemophagocytic lymphohistiocytosis. Pediatr. Blood Cancer. 48:124–131. 10.1002/pbc.21039 16937360

[5] Bergsten, E., A. Horne, M. Aricó, I. Astigarraga, R.M. Egeler, A.H. Filipovich, E. Ishii, G. Janka, S. Ladisch, K. Lehmberg, . 2017. Confirmed efficacy of etoposide and dexamethasone in HLH treatment: Long-term results of the cooperative HLH-2004 study. Blood. 130:2728–2738. 10.1182/blood-2017-06-788349 28935695 PMC5785801

[6] Mahlaoui, N., M. Ouachée-Chardin, G. de Saint Basile, B. Neven, C. Picard, S. Blanche, and A. Fischer. 2007. Immunotherapy of familial hemophagocytic lymphohistiocytosis with antithymocyte globulins: A single-center retrospective report of 38 patients. Pediatrics. 120:e622–e628. 10.1542/peds.2006-3164 17698967

[7] Dufranc, E., A. Del Bello, J. Belliere, N. Kamar, S. Faguer, and TAIDI Toulouse Acquired Immune Deficiency and Infection study group. 2020. IL6-R blocking with tocilizumab in critically ill patients with hemophagocytic syndrome. Crit. Care. 24:166. 10.1186/s13054-020-02878-7 32321563 PMC7175448

[8] Charlesworth, J.E.G., and A. Kavirayani. 2023. Intravenous anakinra for the treatment of haemophagocytic lymphohistiocytosis/macrophage activation syndrome: A systematic review. Eur. J. Haematol. 111:458–476. 10.1111/ejh.14029 37344166

[9] Keenan, C., K.E. Nichols, and S. Albeituni. 2021. Use of the JAK inhibitor ruxolitinib in the treatment of hemophagocytic lymphohistiocytosis. Front. Immunol. 12:614704. 10.3389/fimmu.2021.614704 33664745 PMC7923355

[10] De Benedetti, F., G. Prencipe, C. Bracaglia, E. Marasco, and A.A. Grom. 2021. Targeting interferon-γ in hyperinflammation: Opportunities and challenges. Nat. Rev. Rheumatol. 17:678–691. 10.1038/s41584-021-00694-z 34611329

[11] Locatelli, F., M.B. Jordan, C. Allen, S. Cesaro, C. Rizzari, A. Rao, B. Degar, T.P. Garrington, J. Sevilla, M.C. Putti, . 2020. Emapalumab in children with primary hemophagocytic lymphohistiocytosis. N. Engl. J. Med. 382:1811–1822. 10.1056/NEJMoa1911326 32374962

[12] Vallurupalli, M., and N. Berliner. 2019. Emapalumab for the treatment of relapsed/refractory hemophagocytic lymphohistiocytosis. Blood. 134:1783–1786. 10.1182/blood.2019002289 31537529 PMC8938935

[13] AlAhmari, A., and H. Khogeer. 2023. Successful use of emapalumab in refractory hemophagocytic lymphohistiocytosis in a child with Chédiak-Higashi syndrome: A case report. J. Med. Case Rep. 17:113. 10.1186/s13256-023-03808-1 36978158 PMC10049777

[14] Chandrakasan, S., M.B. Jordan, A. Baker, E.M. Behrens, D. Bhatla, M. Chien, O.S. Eckstein, M.M. Henry, M.L. Hermiston, A.P. Hinson, . 2024. Real-world treatment patterns and outcomes in patients with primary hemophagocytic lymphohistiocytosis treated with emapalumab. Blood Adv. 8:2248–2258. 10.1182/bloodadvances.2023012217 38429096 PMC11117018

[15] Henter, J.I., T. von Bahr Greenwood, and E. Bergsten. 2020. Emapalumab in primary hemophagocytic lymphohistiocytosis. N. Engl. J. Med. 383:596–598. 10.1056/NEJMc2020754 32757534

[16] Lin, H., B.P. Scull, B.R. Goldberg, H.A. Abhyankar, O.E. Eckstein, D.J. Zinn, J. Lubega, J. Agrusa, N. El Mallawaney, N. Gulati, . 2021. IFN-γ signature in the plasma proteome distinguishes pediatric hemophagocytic lymphohistiocytosis from sepsis and SIRS. Blood Adv. 5:3457–3467. 10.1182/bloodadvances.2021004287 34461635 PMC8525230

[17] U.S. Food and Drug Administration . 2018. FDA approves emapalumab for hemophagocytic lymphohistiocytosis. http://www.fda.gov/drugs/fda-approves-emapalumab-hemophagocytic-lymphohistiocytosis (accessed December 8, 2025).

[18] Triebwasser, M.P., D.M. Barrett, H. Bassiri, N. Bunin, C. Elgarten, J. Freedman, A.S. Geera, D. Monos, M.P. Lambert, T. Olson, . 2021. Combined use of emapalumab and ruxolitinib in a patient with refractory hemophagocytic lymphohistiocytosis was safe and effective. Pediatr. Blood Cancer. 68:e29026. 10.1002/pbc.29026 33754483 PMC9269994

[19] Merli, P., I. Caruana, R. De Vito, L. Strocchio, G. Weber, F. Del Bufalo, V. Buatois, P. Montanari, M.G. Cefalo, A. Pitisci, . 2019. Role of interferon-γ in immune-mediated graft failure after allogeneic hematopoietic stem cell transplantation. Haematologica. 104:2314–2323. 10.3324/haematol.2019.216101 30792213 PMC6821635

[20] Verkamp, B., S. Jodele, A. Sabulski, R. Marsh, P. Kieser, and M.B. Jordan. 2024. Emapalumab therapy for hemophagocytic lymphohistiocytosis before reduced-intensity transplantation improves chimerism. Blood. 144:2625–2636. 10.1182/blood.2024025977 39190435

[21] Rybicka-Ramos, M., M. Markiewicz, A. Suszka-Świtek, R. Wiaderkiewicz, S. Mizia, M. Dzierżak-Mietła, and K. Białas. 2022. Profiles of interferon-gamma and interleukin-2 in patients after allogeneic hematopoietic stem cell transplantation. World J. Biol. Chem. 13:72–82. 10.4331/wjbc.v13.i4.72 36187719 PMC9521416

[22] Chellapandian, D., and D. Milojevic. 2023. Case report: Emapalumab for active disease control prior to hematopoietic stem cell transplantation in refractory systemic juvenile idiopathic arthritis complicated by macrophage activation syndrome. Front. Pediatr. 11:1123104. 10.3389/fped.2023.1123104 36891226 PMC9986425

[23] De Benedetti, F., A.A. Grom, P.A. Brogan, C. Bracaglia, M. Pardeo, G. Marucci, D. Eleftheriou, C. Papadopoulou, G.S. Schulert, P. Quartier, . 2023. Efficacy and safety of emapalumab in macrophage activation syndrome. Ann. Rheum. Dis. 82:857–865. 10.1136/ard-2022-223739 37001971 PMC10314091

[24] Debaugnies, F., B. Mahadeb, A. Ferster, N. Meuleman, L. Rozen, A. Demulder, and F. Corazza. 2016. Performances of the H-score for diagnosis of hemophagocytic lymphohistiocytosis in adult and pediatric patients. Am. J. Clin. Pathol. 145:862–870. 10.1093/ajcp/aqw076 27298397

